# Prevention of Mother-to-Child Transmission of HIV/AIDS: Perception of Health Care Workers in Rural Areas of Oyo State

**DOI:** 10.1155/2016/4257180

**Published:** 2016-01-19

**Authors:** Usman Aishat, Ayinde Olubunmi

**Affiliations:** ^1^Department of Community Medicine, LAUTECH Teaching Hospital, PMB 5000, Osogbo, Osun State, Nigeria; ^2^HIV/AIDS Unit, Oyo State Ministry of Health, Ibadan, Nigeria

## Abstract

*Introduction*. Proper implementation of prevention of mother-to-child transmission (PMTCT) services requires adequate knowledge and appropriate attitudes and practices on the part of the health care providers especially in rural areas where access to health care delivery is very limited in Oyo State.* Materials and Methods*. This is a descriptive cross-sectional survey of 350 health care workers in a two-stage sampling technique. Data was obtained using interviewer-administered, pretested, semistructured questionnaires. The data was analyzed using Epi Info software version 7.* Results*. The knowledge of PMTCT of HIV was poor among the health care workers (69.1%). However, more than half (58.3%) had good attitudes towards PMTCT of HIV/AIDS. Predictors of good knowledge of PMTCT were religion [AOR = 1.6, 95% CI (1.1–2.6)], cadre of occupation [AOR = 10.2, 95% CI (2.9–35.1)], and length of service [AOR = 4.3, 95% CI (2.3–19.4)]. Predictors of good attitude towards PMTCT were length of service in the current hospital [AOR = 2.8, 95% CI (1.5–5.2)] and cadre of occupation [AOR = 3.9, 95% CI (1.28–11.9)].* Conclusion*. Despite poor knowledge of PMTCT of HIV/AIDS among the health care workers, the attitude towards PMTCT of HIV/AIDS was good. There is need for the involvement of the stakeholders in bridging the gap between knowledge of and attitude towards prevention of MTCT of HIV/AIDS among health care workers in the rural areas.

## 1. Introduction

Globally, about forty million people are currently living with HIV [1]. This is about one percent of the world population. In 2006, there were about 4.3 million new HIV infections worldwide. Of the total number of AIDS patients globally 48 percent is women with 59 percent in sub-Saharan Africa [2].

It is estimated that more than 60% of Nigerian population lives in rural areas, most of whom are underserved in terms of social amenities [3]. Also, facilities that provide services are concentrated in urban areas particularly in secondary and tertiary health facilities. Universal coverage of highly active ARV treatment (HAART) for HIV-positive pregnant women in Oyo State which is 38% is far below the target of 80% [4].

There is a limited comprehensive intervention package available to postpartum mothers in most health facilities in Oyo State. Interventions on PMTCT were offered in some facilities in Oyo State through the AIDS Prevention Initiative in Nigeria (APIN) project funded by President's Emergency Plan for AIDS Relief (PEPFAR). Child survival is compromised due to limited access to prevention services, in Oyo State.

Prevention of mother-to-child transmission has been considered as not a simple intervention but a comprehensive set of interventions requiring capable health workers. It starts with testing pregnant women for HIV, preferably during their first antenatal visit. When giving the test result, health care workers should provide good counselling, including information about PMTCT options.

The health system should ensure that HIV-positive women receive the PMTCT services that they choose and should provide postnatal care. All along the timeline from finding out their serostatus to getting treatment for HIV-related problems, women and their children should be followed up closely. The need for comprehensive and long-term care for HIV-infected women has become a challenge for health systems, particularly where lack of coordination among different facilities is common [5, 6].

We assessed perceptions of health care workers in rural areas of PMTCT services in Oyo State. The findings of this study should inform the development of a more effective program for the fourth prong of the WHO-recommended comprehensive PMTCT program.

## 2. Methods

This study was a descriptive cross-sectional survey of health care workers in rural PMTCT clinics in Oyo State, southwestern Nigeria. The study was carried out in October 2013. There are three senatorial districts with 120 PMTCT clinics in Oyo State, of which 50 were in rural areas of the state. Selection of respondents was based on their function, position, and experience in the implementation of PMTCT services. Interviewees were first screened to check that they had appropriate positions and at least one year of experience with PMTCT, so that they could provide insightful information. They included doctors, nurses, midwives, community health extension workers, and health assistants. Three hundred and fifty respondents were recruited to the study using a two-stage sampling technique.

Stage 1: The list of all the PMTCT clinics in the rural areas in each senatorial zone was obtained. Simple random sampling was used to select 10 clinics out of the 50 clinics in the rural areas of the state using proportional allocation. Simple random sampling was used to select 5 clinics from Oyo South senatorial zone, 2 clinics from Oyo Central zone, and 3 clinics from Oyo North senatorial zone.

Stage 2: A list of health workers in each PMTCT clinic that was sampled was obtained. Respondents were selected using systematic sampling: the first respondent was randomly selected and every 5th person was chosen as the next respondent until the desired sample size per facility was obtained.

Data was collected with pretested, semistructured interviewer-administered questionnaire. The questionnaire obtained information on the sociodemographic data of the respondents and knowledge and attitude towards the prevention of MTCT of HIV/AIDS. Data was entered into a computer and analyzed using the Epi Info software version 7 [7]. Composite knowledge and attitude scores were computed for PMTCT-related knowledge and attitude by scoring 1 for each correct answer and 0 for an incorrect answer. These scores were then summed up and divided by the total number of test items to arrive at an average knowledge and attitude score per person. Association between sociodemographic characteristics and knowledge of PMTCT of HIV/AIDS was examined using the odds ratio with level of significance set at 5%. Ethical approval was obtained from Oyo State Ministry of Health Ethical Review Committee and written informed consent was obtained from all interviewees, who were invited to participate voluntarily. The interviews were conducted privately and anonymously. There were no refusals.

## 3. Results

Three hundred and fifty questionnaires were administered.  Table 1 shows the background characteristics of the respondents. Mean age of respondents was 40 ± 6.8 years.

Table 2 shows knowledge of health care workers in Oyo State of PMTCT. Overall 37.5% had good knowledge of PMTCT of HIV/AIDS.

Table 3 shows attitude of respondents towards PMTCT. The majority of the respondents (89.7%) agreed that PMTCT is an important program. Almost all the respondents (91.4%) agreed that there was not enough time to render PMTCT services.

Table 4 shows the association between background characteristics of respondents and knowledge level of PMTCT of HIV/AIDS. The bivariate and multivariate analysis show that marital status, sex, and time spent in the current hospital had no significant association with PMTCT knowledge. Health care workers who were Christian were two times more likely to be knowledgeable about PMTCT than the Muslims [AOR = 1.6, 95% CI (1.3–7.8)]. Doctors and nurses were ten times more likely to be knowledgeable about PMTCT of HIV/AIDS than health assistants [AOR = 10.2, 95% CI (3.0−35.1)]. Health care workers who had spent 5 years and more in the hospital were four times more likely to be knowledgeable about PMTCT of HIV/AIDS than those who had spent less than 5 years.

Table 5 shows association between background characteristics of respondents and their attitude towards PMTCT. Health care workers who had spent between 5 years and above were three times more likely to have good attitude towards PMTCT of HIV/AIDS than those who had spent less than 5 years in the current hospital [AOR = 2.7, 95% CI (1.5–5.2)]. Community health officers/community health extension officers were four times more likely to have good attitude towards PMTCT of HIV/AIDS than health assistants [AOR = 3.9, 95% CI (1.3–11.9)].

## 4. Discussions

Health care workers had poor knowledge about PMTCT of HIV/AIDS. Though the knowledge about PMTCT was poor among the respondents, the majority have a good attitude towards PMTCT. This is in contrast to a study done in Botswana, where having a high PMTCT knowledge score was a major factor associated with good attitude towards PMTCT. Also a study carried out in Port Harcourt, Nigeria, reported that 89.3% of general medical practitioners agreed that their knowledge of, attitude towards, and practice of PMTCT of HIV/AIDS were deficient [8]. In a study conducted on 53 health care workers in Vietnam identified inadequate knowledge and skills regarding PMTCT among health care workers were one of the factors affecting the delivery of PMTCT services [9].

Religion is associated with good knowledge of prevention of MTCT of HIV/AIDS. Being Christian has significant association with good knowledge about PMTCT. This finding is also in line with a study from Osogbo, Nigeria, where religion was associated with knowledge of PMTCT of HIV/AIDS [10]. Also a study from Ghana revealed that Christians had good knowledge of PMTCT compared to the Muslims [11]. This finding may be due to the fact that religious leaders are getting involved in campaigns against HIV infection. Studies have showed that religious organizations are recognized for playing an important role in preventing new infections in Uganda [12, 13]. Nearly all of its major religious institutions, both Islamic and Christian, have been actively engaged in the country's struggle with HIV/AIDS [12]. Many nongovernmental organizations do plenty of collaborative work with religious organizations in Nigeria, all in a bid to increase awareness of MTCT and its prevention. Unfortunately however, many faith-based organizations see immoral behavior as being the cause of the HIV/AIDS epidemic and they therefore decline involvement in preventive and intervention programs [12].

Doctors and nurses were found to have good knowledge of PMTCT of HIV/AIDS. This is in line with findings of Nneka et al. (2007) where they reported doctors and nurses were more knowledgeable than paramedics [14]. A study from Vietnam revealed that oftentimes training was provided to doctors on HIV/AIDS and PMTCT [9]. This finding may be due to the training doctors and nurses underwent which gives background information on HIV/AIDS better than other cadres of health care workers. It may also be as a result of previous contact and management of HIV-positive pregnant women by doctor and nurses.

Length of services was also associated with good knowledge and attitude towards PMTCT of HIV/AIDS. It was found that health care workers who had spent five years and above were more knowledgeable about prevention of MTCT. This may be due to repetitive exposure to HIV patients by health care workers thereby acquiring more knowledge through on-job training. There is possibility that HIV training can significantly improve such knowledge regarding HIV transmission and prevention. However, training alone may not have provided adequate information in these areas, since knowledge scores for some items were low for all workers. These results suggest that all health care workers may benefit from increased HIV/AIDS education in the context of PMTCT that recognizes existing gaps and prevent infection.

Therefore, thorough training of health personnel in all aspects of mother-to-child transmission, feeding recommendations and options, and further preventive measures is indispensable.

The finding from this present study revealed that community health officers/extension workers had good attitude towards prevention of mother-to-child transmission of HIV/AIDS. A study from Maiduguri, Nigeria, revealed that nurses had poorer attitude towards PMTCT than doctors and other paramedical staff [14]. This could be due to the fact that the majority of the health care workers in the rural areas were community health officers/extension workers and were already used to delivering PMTCT services. Studies have shown that less specialized cadres of health care workers are capable of effective delivery of HIV care with comparable and even better outcomes as physicians [15]. Length of services was also associated with good attitude towards PMTCT of HIV/AIDS. This may be as a result of on-job training and practice.

## 5. Conclusion and Recommendations

Knowledge and attitudes on PMTCT amongst health care workers show that there was poor knowledge although the attitude was acceptable. Without further training and increase in staffing levels, the quality and access to PMTCT services will likely be negatively impacted. A wide gap exists between the knowledge of and attitude towards PMTCT of HIV/AIDS.

Health promotional activities and behavioral change through effective communication routes such as refreshers course, trainings, and workshops should be promoted. This would take targeted health education messages beyond impacting knowledge. More elaborate studies should be carried out in this field in other parts of Nigeria in order to ensure improvement in knowledge and behavior.

## Figures and Tables

**Table 1:** Background characteristics of respondents.

Characteristics	Frequency (%)
Length of service (years)	
1–5	38 (10.9)
6–10	103 (29.4)
11–15	119 (34.0)
16–20	55 (15.7)
≥21	35 (10.0)
Mean length of service	12.66 ± 6.65
Age group	
≤29	27 (7.7)
30–34	26 (7.4)
35–39	80 (22.9)
40–44	121 (34.6)
≥45	96 (27.4)
Mean age ± standard deviation	40 ± 6.8 years
Sex	
Male	61 (17.4)
Female	289 (82.6)
Level of education	
University	99 (28.3)
College	76 (21.7)
Secondary	35 (10.0)
Primary	140 (40.0)
Cadre of workers	
Doctor	16 (4.6)
Nurse	73 (20.9)
CHO	30 (8.6)
CHEW	33 (9.4)
Health assistant	198 (56.6)
Religion	
Christianity	227 (64.9)
Islam	123 (35.1)
Marital status	
Married	328 (93.7)
Widowed	10 (2.9)
Single	12 (3.4)

**Table 2:** Respondents knowledge of PMTCT of HIV/AIDS (*n* = 350).

Variables	Correct response (%)	Wrong response (%)
HIV Prevalence in general population	170 (48.6)	180 (51.4)
HIV Prevalence in women	144 (41.1)	206 (58.9)
Full meaning of acronyms of PMTCT	153 (43.7)	197 (56.3)
Vertical transmission risk	226 (64.6)	124 (35.4)
Transmission risk with breastfeeding	256 (73.1)	94 (26.9)
Awareness of guidelines on PMTCT	100 (28.6)	250 (71.4)
When to initiate PMTCT drugs	123 (35.1)	227 (68.9)

**Table 3:** Attitude of health care workers towards PMTCT of HIV/AIDS (*n* = 350).

Variables	Number (percentage)		
	Disagree	Indifferent	Agree
PMTCT program is very important	24 (6.9)	29 (8.3)	297 (84.9)
Providing PMTCT stops us from providing good ANC care	257 (73.4)	29 (8.3)	64 (18.5)
I am scared to deliver HIV-positive women because of fear of infection	157 (44.9)	12 (3.4)	181 (51.7)
HIV-infected pregnant women must be delivered by skilled personnel	44 (12.6)	72 (20.6)	234 (66.9)
There is not enough time to give to PMTCT services	18 (5.1)	12 (3.4)	320 (91.4)
HIV-infected woman may not breastfeed her child if there is risk of infection	97 (27.7)	64 (18.3)	189 (54.0)

**Table 4:** Association between background characteristics of respondents and good knowledge of PMTCT of HIV/AIDS.

Characteristics	Crude odds ratio	Adjusted odds ratio	*p* value
Age (years)			
<40	5.7 (0.4–5.3)	2.3 (0.3–14.0)	0.30
≥40	Ref.		
Sex			
Male	Ref.	Ref.	
Female	3.1 (1.6–7.5)	1.6 (0.9–7.9)	0.12
Level of education			
Less than secondary	6.3 (0.7–11.9)	4.3 (0.7–7.5)	0.67
Secondary and above	Ref.	Ref.	
Marital status			
Single	Ref.	Ref.	
Married	3.9 (0.8–4.6)	2.9 (0.4–4.0)	0.27
Religion			
Christianity	2.1 (1.1–4.8)^*∗*^	1.6 (1.3–7.8)	0.01^*∗*^
Islam (ref.)	Ref.		
Cadre of occupation			
Doctor/nurse	10.8 (2.7–43.6)	10.2 (3.0–35.1)^*∗*^	0.00^*∗*^
CHO/CHEW	0.4 (0.3–2.5)	0.9 (0.2–4.1)	
Health assistant	Ref.	Ref.	
Time spent in current hospital			
<5 years	Ref.	Ref.	
≥5 years	0.9 (0.3–2.9)	4.3 (2.3–19.4)^*∗*^	0.00^*∗*^

^*∗*^ *p* < 0.05 level of statistical significant.

**Table 5:** Association between background characteristics of respondents and good attitude towards PMTCT of HIV/AIDS.

Characteristics	Crude odds ratio	Adjusted odds ratio	*p* value
Age (years)			
<40	0.7 (0.4–1.3)	1.0 (0.6–1.8)	0.24
≥40	Ref.	Ref.	
Sex			
Male	Ref.	Ref.	
Female	2.3 (0.9–9.2)	1.9 (0.8–6.5)	0.62
Level of education			
Less than secondary	3.2 (0.7–3.9)	2.9 (0.9–6.3)	0.12
Secondary and above	Ref.	Ref.	
Marital status			
Single	Ref.	Ref.	
Married	1.8 (0.2–9.1)	3.8 (0.9–4.6)	0.23
Length of service (years)			
<5 years	Ref.	Ref.	
≥5 years	3.2 (1.5–6.5)^*∗*^	2.7 (1.5–5.2)^*∗*^	0.02^*∗*^
Religion			
Christianity	1.5 (0.4–3.2)	2.4 (0.3–3.8)	0.25
Islam	Ref.	Ref.	
Cadre of occupation			
Doctor/nurse	3.7 (0.9–5.6)	0.5 (0.1–2.4)	0.01^*∗*^
CHO/CHEW	1.4 (0.8–9.7)	3.9 (1.3–11.9)^*∗*^	
Health assistant	Ref.	Ref.	

^*∗*^ *p* < 0.05 level of statistical significant.
